# Roux-En-Y Gastric Bypass Vs. Sleeve Gastrectomy: Balancing the Risks of Surgery with the Benefits of Weight Loss

**DOI:** 10.1007/s11695-016-2265-2

**Published:** 2016-06-24

**Authors:** Corey J. Lager, Nazanene H. Esfandiari, Angela R. Subauste, Andrew T. Kraftson, Morton B. Brown, Ruth B. Cassidy, Catherine K. Nay, Amy L. Lockwood, Oliver A. Varban, Elif A. Oral

**Affiliations:** 1 Department of Internal Medicine, Division of Metabolism, Endocrinology and Diabetes (MEND), University of Michigan, 1000 Wall Street, Ann Arbor, MI USA; 2 Division of Endocrinology, University of Mississipi, Jackson, MS USA; 3 Department of Biostatistics, School of Public Health, University of Michigan, Ann Arbor, 48109 MI USA; 4 Division of Minimally Invasive Surgery, Department of Surgery, University of Michigan, Ann Arbor, 48109 MI USA

**Keywords:** Obesity, Bariatric surgery, Weight loss, Surgical complications, Metabolism

## Abstract

**Background:**

The purpose of the study was to compare weight loss, metabolic parameters, and postoperative complications in patients undergoing Roux-en-Y gastric bypass (GB) and sleeve gastrectomy (SG).

**Methods:**

We retrospectively studied 30-day postoperative complications as well as change in weight, blood pressure, cholesterol, hemoglobin, hemoglobin A1C, and creatinine from baseline to 2, 6, 12, and 24 months postoperatively in 383 patients undergoing GB and 336 patients undergoing SG at the University of Michigan from January 2008 to November 2013. For a study population which typically has high attrition rates, there were excellent follow-up rates (706/719 at 2 months, 566/719 at 6 months, 519/719 at 12 months, and 382/719 at 24 months).

**Results:**

Baseline characteristics were similar in both groups except for higher weight and BMI in the SG group. The GB group experienced greater total body weight loss at 6, 12, and 24 months (41.9 vs. 34.6 kg at 24 months, *p* < 0.0001). Excess weight loss was 69.7 and 51.7 % following GB and SG respectively at 24 months (*p* < 0.0001). BP improved significantly in both groups. Surgical complication rates were greater after GB (10.1 vs. 3.5 %, *p* = 0.0007) with no significant difference in life-threatening or potentially life-threatening complications.

**Conclusions:**

Weight loss was greater following GB compared to SG at 2 years. The risk for surgical complications was greater following GB. Surgical intervention should be tailored to surgical risk, comorbidities, and desired weight loss.

**Electronic supplementary material:**

The online version of this article (doi:10.1007/s11695-016-2265-2) contains supplementary material, which is available to authorized users.

## Introduction/Purpose

Obesity afflicts tens of millions of Americans and the number of Americans with morbid obesity is still on the rise [1]. Given the lack of effective preventive and medical strategies to combat obesity, surgical approaches have emerged as favorable treatment options for patients facing this chronic and distressing health problem although the optimal weight loss surgery remains controversial.

Laparoscopic sleeve gastrectomy (SG) is relatively new as a stand-alone procedure for weight loss in patients with morbid obesity, with coverage first provided in the USA by the Center for Medicare and Medicaid Services in 2012, and even then only on a regional basis [2]. Laparoscopic gastric bypass (GB) has been available significantly longer with well-documented weight loss and resolution of obesity-related comorbidities [3]. Comparisons of the two procedures have primarily been in the form of observational studies mostly with <30 % of retention rate by 24 months. Several RCTs with sample size less than 50 patients have demonstrated substantial weight loss with both procedures, but no significant difference between them [4–7]. Two larger RCTs (*n* = 72 and 217) with 1-year follow-up and one RCT (*n* = 60) with 3-year follow-up also demonstrated no significant difference [8–10]. Two larger RCTs (*n* = 60 and 100) and a meta-analysis of RCTs demonstrated significantly greater weight loss with GB, although this was a secondary outcome in both primary studies [11–13]. Large observational studies and two meta-analyses echo these results, with some showing similar weight loss and some showing both greater weight loss and resolution of obesity-related comorbidities with GB [14–22].

As the benefit of weight loss must be balanced against potential risks of surgery, it is important to consider both immediate and chronic complications following the different types of obesity surgeries. Large observational studies and RCTs (*n* > 50) as well as a meta-analysis show either no differences between procedures [9] or a greater rate of one or more of the following for GB: minor complications, longer hospital stay, and longer operative time [8, 10, 12, 14, 16, 18–24].

In the longer term, obesity surgery can lead to malabsorption of micronutrients such as vitamins and minerals [25] and a loss of lean mass [26]. Anemia may develop as a result of micronutrient malabsorption [25]. Gastric bypass and historical procedures have demonstrated an increased risk of urolithiasis and a small risk of acute kidney injury [27].

Given the relatively new emergence of SG as a stand-alone procedure, and relative paucity of data on well-established cohorts captured from the real-world settings with a high rate of retention, we decided to capitalize on the longitudinal follow-up available at our institution’s Post-Bariatric Surgery Clinic. We recognized the changing patterns in the choice of surgery by both our patients and surgeons and wanted to explore whether the outcomes of these two surgeries are really comparable in our cohort. Therefore, the current retrospective study was performed to gain further insight into the differences between GB and SG with respect to weight loss and postoperative complications. In addition, we were interested in comparison of blood pressure and HbA1c as a surrogate for metabolic improvement. We further explored changes in creatinine as a marker of glomerular hyperfiltration and lean body mass [28, 29]. Lastly, development of anemia in the first 2 years following obesity surgery was explored as an early surrogate for long-term complications.

## Materials and Methods

### Patients

All patients over 17 years of age undergoing GB or SG for obesity at the University of Michigan between January 2008 and November 2013 were included in our retrospective study. All patients had either body mass index (BMI)>40 kg/m^2^ or BMI >35 kg/m^2^ with an obesity-related comorbidity and had previously been unsuccessful with medical treatment for obesity, consistent with Medicare and Medicaid requirements for insurance coverage of bariatric surgery. Exclusion criteria included previous bariatric surgery or the inability to complete the surgery related to unexpected operative finding. We identified 383 patients for GB (305 female) and 336 patients for SG (259 female) who met these criteria and were included for analysis. Institutional review board approval was obtained.

### Data Collection

The University of Michigan Adult Bariatric Surgery Program is a multidisciplinary approach to weight loss [30]. Patients presenting for evaluation for bariatric surgery first undergo rigorous medical, dietary, and psychological evaluation. After surgery, patients follow up with the surgeon and the dietitians at 2 weeks and at 2 months postoperatively. Subsequent follow-up is with the endocrinologist and the dietitian at the Post-Bariatric Surgery Clinic, where patients are asked to present at 6, 12, and 24 months (as well as yearly thereafter) postoperatively to evaluate for and treat vitamin deficiencies, weight regain, and obesity-related comorbidities. Patients are asked to take a standard supplement regimen that includes vitamin B12, vitamin D, calcium citrate, and multivitamin with or without iron [31].

Retrospective electronic medical review was performed, and data was abstracted from visits that occurred preoperatively (within 60 days prior to surgery) and postoperatively at 2 months ±2 weeks, 6 months ±1 month, 12 months ±2 months, and 24 months ±3 months. Baseline demographic characteristics collected included sex and age. Data collected at baseline and follow-up, when available, included weight, height, BMI, blood pressure, creatinine, glucose, HbA1c, and hemoglobin. We also collected 25-hydroxy vitamin D, vitamin B12, folic acid, vitamin A, vitamin B1, total cholesterol, HDL, LDL, and triglyceride levels. Excess body weight loss (%) was calculated by dividing total weight loss by the difference between actual body weight and ideal body weight, which is the weight at a BMI of 25 kg/m^2^ for each patient.

Thirty-day surgical complications were collected by electronic medical record review. Complications are graded from I–III (I is non-life-threatening, II is potentially life-threatening, III is life-threatening and associated with residual and lasting disability) based on definitions provided by the Michigan Bariatric Surgery Collaborative reporting dictionary discussed in detail elsewhere [32]. Grade I complications include surgical site infection requiring antibiotics and/or wound opening, hospital-acquired infections requiring antibiotics, hemorrhage requiring endoscopy or blood transfusion of less than or equal to four units, or anastomotic stricture requiring dilatation. Grade II complications include abdominal abscess requiring drainage or reoperation, bleeding requiring transfusion greater than four units, respiratory failure requiring 2–7 days of intubation, renal failure requiring in-hospital dialysis, venous thromboembolism (VTE), or reoperation for leak, bowel obstruction, hernia, wound infection, or dehiscence. Grade III complications include myocardial infarction, cardiac arrest, acute renal failure requiring long-term dialysis, respiratory failure requiring >7 days on ventilator or tracheostomy, or death. Utilizations assessed include reoperation, length of stay greater than 4 days, readmission, and emergency department visit.

### Statistical Analysis

Continuous data are reported as mean ± standard deviation unless otherwise specified in figures where standard error of the mean (SEM) may be shown. Complication and utilization data are reported as frequency (percent). Changes in laboratory data, weight, BMI, blood pressure, hemoglobin, creatinine, and HbA1c were compared between procedures by the Mann-Whitney-Wilcoxon rank sum test. Utilizations and surgical, medical, and graded complications were analyzed by fitting a multivariable logistic model for each complication or utilization in order to compare the complication rates by procedures while adjusting for baseline characteristics including age, gender, weight, tobacco history, mobility limitations, and medical history (lung disease, cardiovascular disease, hypertension, hyperlipidemia, gastroesophageal reflux, peptic ulcer disease, cholelithiasis, urinary incontinence, renal failure, diabetes, liver disease, history of VTE, sleep apnea, musculoskeletal disorders, history of hernia repair, and psychological disorders). Any adverse event is defined as the occurrence of any complication, utilization, or death. *P* values are reported for each test employed. Analyses were done using SAS 9.4 64-bit Windows.

## Results

### Demographic Data, BMI, Blood Pressure, Serum Values

The total study population included 719 patients (564 females, 78 %) with mean age of 44 years and baseline BMI of 48.4 kg/m^2^. Baseline characteristics (Table 1) were similar between the two groups except BMI and weight, which were significantly greater in the SG group.

**Table 1:** Baseline characteristics

	GB mean (SD)	GB *n*	SG mean (SD)	SG *n*	*p* value
Female (%)	80	383	77	336	0.17
Age (years)	43.7 (11.2)	383	45.1 (10.7)	336	0.13
Weight (kg)	133.1 (26.9)	383	141.2 (29.1)	336	<0.0001
BMI (kg/m^2^)	47.2 (7.8)	383	49.7 (8.8)	336	<0.0001
Systolic BP (mmHg)	137.2 (18.5)	372	137.7 (18.5)	313	0.46
Hemoglobin A1C (%)	6.7 (1.4)	149	6.7 (1.3)	123	0.86
Vitamin D (ng/mL)	29.6 (14.6)	268	29.2 (14.0)	270	0.92
Vitamin B12 (pg/mL)	538.7 (233.1)	168	587.5 (287.9)	101	0.20
Hemoglobin (g/dL)	13.3 (1.4)	383	13.3 (1.2)	330	0.19
Creatinine (mg/dL)	0.84 (0.19)	379	0.84 (0.22)	330	0.14
Total cholesterol (mg/dL)	180.1 (33.9)	52	174.4 (34.3)	55	0.49

*GB* Roux-en-Y gastric bypass, *SD* standard deviation, *SG* sleeve gastrectomy

### Availability of Data for Outcomes of Interest

Three hundred eighty, 304, 256, and 173 patients in GB and 326, 262, 253, and 209 patients in SG returned for their 2, 6, 12, and 24 months visits respectively and provided data on weight loss and change in BMI. Full data availability within windows defined above for other variables is provided in Table 2.

**Table 2:** Data availability

Months	2	6	12	24
GB number of patients (% follow-up)				
Weight/BMI	380 (99 %)	304 (79 %)	256 (67 %)	173 (45 %)
SBP	362 (97 %)	294 (79 %)	246 (66 %)	173 (47 %)
Hemoglobin	380 (99 %)	233 (61 %)	179 (47 %)	100 (26 %)
Creatinine	184 (49 %)	191 (50 %)	140 (37 %)	124 (33 %)
SG number of patients (% follow-up)				
Weight/BMI	326 (97 %)	262 (78 %)	253 (75 %)	209 (62 %)
SBP	302 (97 %)	242 (77 %)	223 (71 %)	191 (61 %)
Hemoglobin	327 (99 %)	193 (59 %)	146 (44 %)	126 (38 %)
Creatinine	184 (56 %)	191 (58 %)	140 (42 %)	124 (38 %)

*GB* Roux-en-Y gastric bypass, *SG* sleeve gastrectomy

### Change in Operative Procedure Frequency

We noticed a change in the total number of bariatric procedures as well as type of operations performed at our institution within the time frame of interest. In 2008, all 63 operations performed were GB. The total number of procedures and the GB% reported in parentheses in 2009, 2010, 2011, 2012, and 2013 respectively are 111 (87 %), 117 (60 %), 156 (44 %), 144 (37 %), and 128 (24 %).

### Weight Loss and Metabolic Parameters

**Fig. 1:**
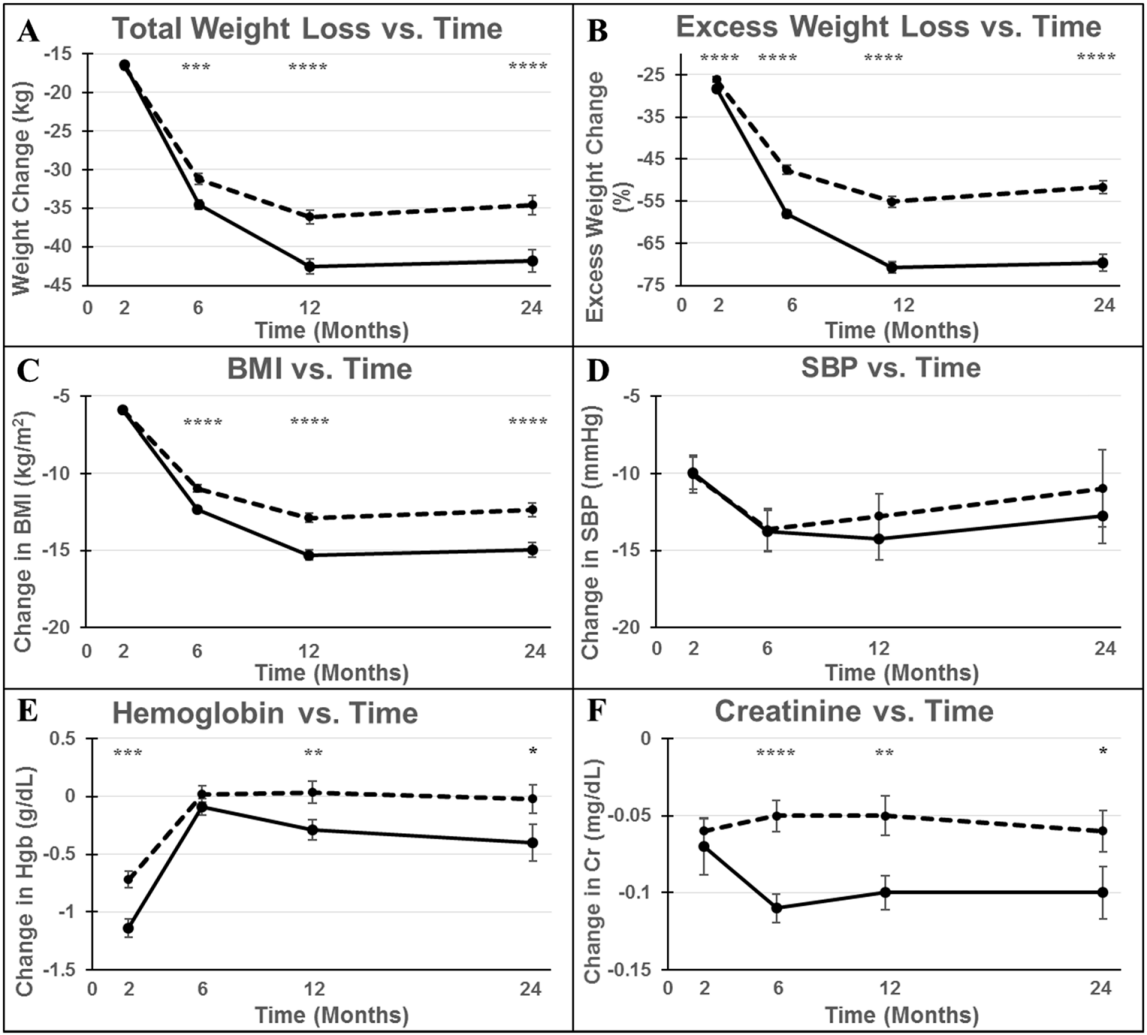
Comparison of metabolic outcomes of sleeve gastrectomy (SG, *dotted line*) vs. Roux-en-Y gastric bypass (GB, *solid line*). All graphs show the change in the specified variable over time. *Error bars* represent SEM. *BMI* body mass index, *SBP* systolic blood pressure, *Hgb* hemoglobin, *Cr* creatinine. *P < 0.05; **P < 0.01; ***P < 0.001; ****P < 0.0001 for comparison between procedures

Change in weight over time utilizing three different indices is shown in Fig. 1 a–c: change in weight (a), excess body weight (b), and BMI (c). The GB group experienced greater and more rapid weight loss, losing 42.6 kg of total body weight by 12 months vs. 36.1 kg in the SG group (*p* < 0.0001). This difference was preserved at 24 months (41.9 vs. 34.6 kg, respectively, *p* < 0.0001). BMI similarly improved more significantly in GB with a decrease of 15.3 kg/m^2^ vs. 12.9 kg/m^2^ with SG at 12 months and 15.0 kg/m^2^ at 24 months with GB vs. 12.4 kg/m^2^ with SG (*p* < 0.0001 and *p* = 0.0001 respectively). Excess body weight loss was significantly greater with GB at all time points (70.8 vs. 55.2 % at 12 months, 69.7 vs. 51.7 % at 24 months, *p* < 0.0001 at 12 and 24 months).

Systolic blood pressure (Fig. 1d) improved similarly and highly significantly in both groups with a decrease of 12.8 mmHg after GB (*p* < 0.0001) and 11.0 mmHg after SG (*p* < 0.0001) at 24 months (*p* = 0.65 for intergroup comparison). The improvement in HbA1c was also greater with GB compared to SG as shown in Supplementary Fig. 1; however, data were available for only 10 to 15 % of the patients within the time points specified, providing data on 25 to 60 % of individuals with diabetes at any time point, decreasing the strength of statistical comparisons performed. Lipid levels were not reported due to insufficient data availability.

### Hemoglobin and Creatinine

Both groups experienced a drop in hemoglobin at 2 m, with GB patients displaying a significantly greater decrease compared to SG patients (1.14 vs. 0.72 g/dL, *p* = 0.0003, Fig. 1e). The fall in hemoglobin was not protracted and improved nearly back to baseline by 6 m. The GB group displayed a fall in serum creatinine (Fig. 1f) that reached statistical difference at 2 months and was sustained for long-term while the SG group experienced a less remarkable decrease in serum creatinine (0.10 mg/dL following GB vs. 0.05 following SG, *p* = 0.002 at 12 months).

### Complications

**Fig. 2:**
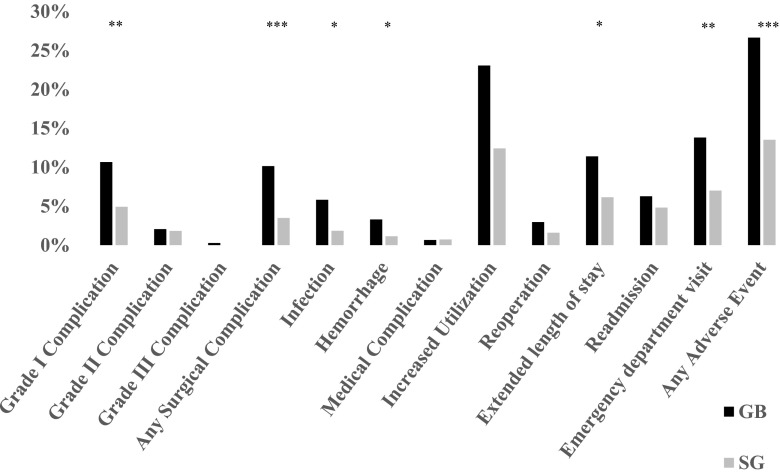
Comparison of utilization and graded surgical and medical complications of sleeve gastrectomy (SG, *gray*) and Roux-en-Y gastric bypass (GB, *black*). *P < 0.05; **P < 0.01; ***P < 0.001; ****P < 0.0001 between procedures

Rates of 30-day complications and adverse events (Fig. 2) were generally greater following GB compared with SG. Rates of overall surgical complications were greater in the GB group (10.2 vs. 3.5 % with SG, *p* = 0.0007). This was primarily driven by infectious complications (5.8 vs. 1.8 %, respectively, *p* = 0.01) and hemorrhage (3.3 vs. 1.1 %, *p* = 0.04). There were no significant differences between groups with respect to leak/perforation or obstruction, which were rare in both groups. Medical complications were likewise rare and did not differ between groups. Grade I complications were significantly greater in the GB group (10.7 vs. 4.9 %, *p* = 0.004). Grade II and III complications were rare and did not differ between groups. Overall increased utilization was not significantly different between groups (23.1 vs. 12.4 %, *p* = 0.22); however, visits to the emergency department (13.8 vs. 7.0 %, *p* = 0.002) and rates of extended length of stay (11.4 vs. 6.2 %, *p* = 0.01) were more frequent in the GB group. There were no significant differences between rates of reoperation (3.0 vs. 1.6 % with GB and SG, respectively, *p* = 0.23) and rates of readmission (6.3 vs. 4.8 % with GB and SG, respectively, *p* = 0.36). There were no deaths observed.

### Effect of Comorbidities on Weight Loss and Metabolic Outcomes

A general linear model was fit to the dataset to adjust for preoperative comorbidities including diabetes, hyperlipidemia, cardiovascular disease, and gender. The adjustments did not change the degree of statistical significance for changes observed in body weight, BMI, percent excess weight loss, or blood pressure. Given the number of available observations for hemoglobin, creatinine, and HbA1c, we did not perform risk adjustments for these parameters.

### Impact of Surgery Date and Preoperative Weight on Weight Loss and Metabolic Outcomes

To reduce the risk of confounding due to the different baseline characteristics in our study population, reanalysis was done for patients who had surgery after December 2010. This cohort (mean age 44.5 years, 79.0 % female, mean BMI 48.2 kg/m^2^) demonstrated no significant differences in baseline characteristics between the two groups, including weight and BMI, and underwent surgery at a time when similar numbers of GB and SG were performed (Supplementary Table 1). These separate analyses showed similar trends to the data in Fig. 1 and are displayed in Supplementary Fig. 2.

## Discussion

This observational retrospective cohort study is one of the largest single-center studies to examine outcomes including weight loss, limited markers of obesity-related comorbidities, and surgical complications to determine the relative risks and benefits of GB and SG. We have seen that patients lose a larger amount of weight and sustain the difference up to 2 years with GB compared to SG.

Since late October 2013, SG has been the predominant bariatric surgery procedure at our institution. There is a similar trend across all weight loss surgery centers in the USA [33]. However, our results, and those of other similar studies [4–13, 15–19, 21–24, 27], suggest a greater weight loss and/or improvement in obesity-related comorbidities including diabetes and hyperlipidemia, as well as similar rates of serious (grades II–III) complications with GB [14]. The established drawbacks of GB are the increase in grade I complications, extended length of stay, and emergency department visits.

Greater weight loss and improvements in metabolic parameters are hypothesized to be related to both bypass of the proximal small intestine (upper intestinal hypothesis), which alone has been shown in rat models to improve glucose homeostasis, as well as increased nutrient delivery to the distal small intestine. Additional studies in rodents have shown the latter mechanism alone to independently increase glucagon-like peptide-1 (GLP-1) and peptide YY to improve glucose homeostasis [34]. Somewhat unexpectedly, similar hormonal changes following SG were seen in a small study by Peterli et al. in 2012 [5]. Possible explanations included more rapid gastric emptying following SG and elevations in cholecystokinin (CCK) causing downstream hormonal effects, including increased GLP-1. More recent focus has been directed to changes in bile acids as well as gut microbiome providing a link between metabolic amelioration and obesity surgeries [35].

The greater decrease in creatinine following GB compared with SG is a little bit more complex to interpret, as a decrease in creatinine has been associated with both improvement in obesity-related glomerular hyperfiltration and a decrease in lean body mass [28, 29]. The greater decrease in hemoglobin at the early postoperative visit is consistent with the increased rate of hemorrhage following GB, but it is reassuring that hemoglobin returned close to baseline thereafter.

While originally we wanted to evaluate the changes in vitamin levels in both groups, data availability was limited, and therefore we will refrain from comments regarding the effect of the surgical procedures on the various vitamin levels. We will be evaluating these in future studies. It should be noted that both groups have been undergoing aggressive surveillance and supplementation regimens consistent with accepted standards of care [31].

There are several limitations that should be considered for interpretation of our results. First, the study was comprised of retrospective data analyses of clinical follow-up, potentially allowing for selection and other biases. However, groups were similar at baseline in all measured variables except for increased weight and BMI in the SG group. This difference is likely explained by insurance coverage for SG only with BMI >50 kg/m^2^ for the earlier part of the study [2]. In addition, the known higher operative risk in patients with greater baseline BMI [36] and lower operative risk with SG compared with GB [14] likely caused patients and surgeons to favor SG in patients with greater BMI.

The modifying effect of higher baseline BMI in the SG group on weight loss depends on the metric used to measure weight loss. Studies have demonstrated that even when looking at a single weight loss surgery such as GB, greater preoperative BMI is associated with decreased *excess* body weight loss [37], however with no significant difference in *total* body weight loss [38]. Our own analysis confirmed that weight loss differences at 6–24 months remained highly significant when adjusted for baseline BMI. Since the GB and SG groups had a similar baseline BMI after 2011, a repeat analysis excluding data from 2008 to 2010 was performed which demonstrated significant differences in both total and excess weight loss at 6–24 months. This suggests that the greater total body weight loss following GB observed in our study after 6 months is not explained by the differences in baseline BMI.

A second important limitation of the study was that available biochemical data was incomplete at 2, 6, 12, and 24 months data points as some patients did not return to clinic for blood draws or returned outside of the allowable time range for our analyses. Finally, as this study was performed at a single tertiary care referral center, results may not be generalizable to other institutions.

Strengths of the study include large sample size, sequential patient inclusion which makes the results more generalizable to all patients in comparison to the more stringent requirements for RCTs, and moderately long-term follow-up of 2 years. In addition, due to the unique timespan of the study providing a more balanced evaluation of the procedures, the dataset includes roughly an even distribution of patients between the two surgical procedures.

Now that our cohort (whom we would like to call Michigan Post Bariatric Surgery Cohort, MI-BASiC) is established, we are planning to follow our patients for a longer term to determine if the differences we have observed in weight loss will be sustained over 5 years. Most studies that reported data beyond 5 years have high attrition rates [20, 24]. Even if the gap of metabolic control and weight loss eventually closes, it is possible to observe a “legacy” effect of a favorable state, the so-called metabolic memory, which may translate into meaningful outcomes as has been observed from UKPDS or DCCT/EDIC [39, 40].

## Conclusion

Our results raise the possibility that the obesity surgery field has moved to SG predominance too quickly, ahead of full characterization of observed benefits. Further research is needed to evaluate the current trend of SG being the surgery of choice at most bariatric surgery centers, with long-term follow-up and subgroup analyses needed to provide more precise, personalized information to patients seeking weight loss surgery.

## Electronic supplementary material

ESM 1 (PDF 138 kb)
