# Supplementary material for: Roux-En-Y Gastric Bypass Vs. Sleeve Gastrectomy: Balancing the Risks of Surgery with the Benefits of Weight Loss
Source: Obes Surg. 2016 Jun 24;27(1):154–61. doi: 10.1007/s11695-016-2265-2 (PMC5187368; doi:10.1007/s11695-016-2265-2)
Supplement: Supplementary file 1 — (PDF 138 kb) [file 11695_2016_2265_MOESM1_ESM.pdf]

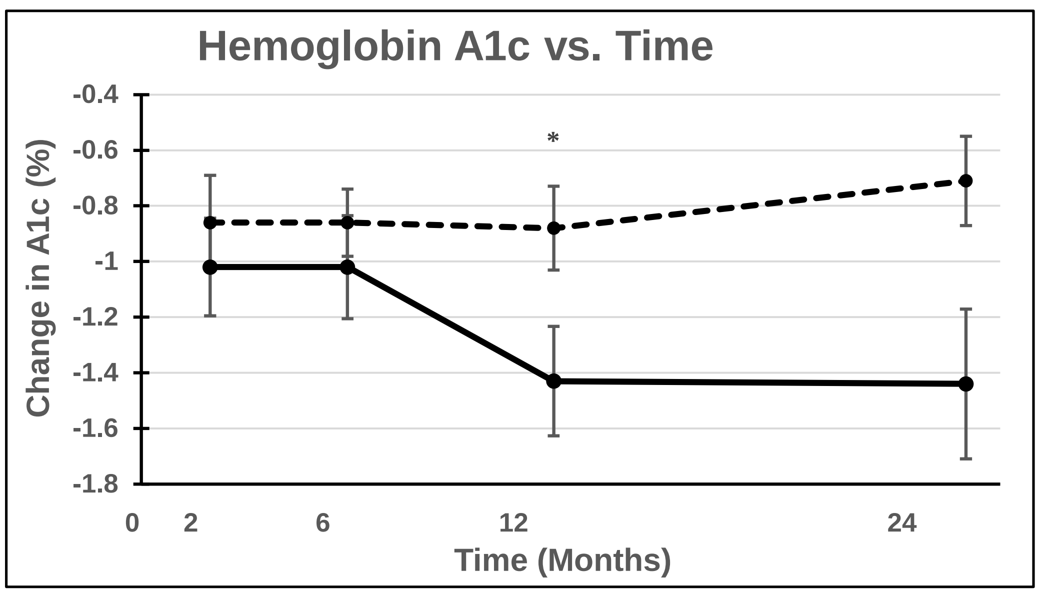

Supplementary Figure 1. Comparison of the change in hemoglobin A1C following sleeve gastrectomy (SG, dotted line) vs. Roux-en-Y gastric bypass (GB, solid line). Diabetes was present in 40% of GB patients and 31% of SG patients at baseline with mean HbA1c of 6.7% ± 1.4% and 6.7% ± 1.3% respectively (NS). Data were available for 10-15% of all patients, including diabetic and non-diabetic patients, at specified time points (n = 38, 57, 45, and 26 for GB and 34, 58, 47, and 28 for SG at 2, 6, 12, and 24m respectively). Error bars represent standard error of the mean. For comparison between procedures: *, P < 0.05; **, P < 0.01; ***, P < 0.001; ****, P < 0.0001.

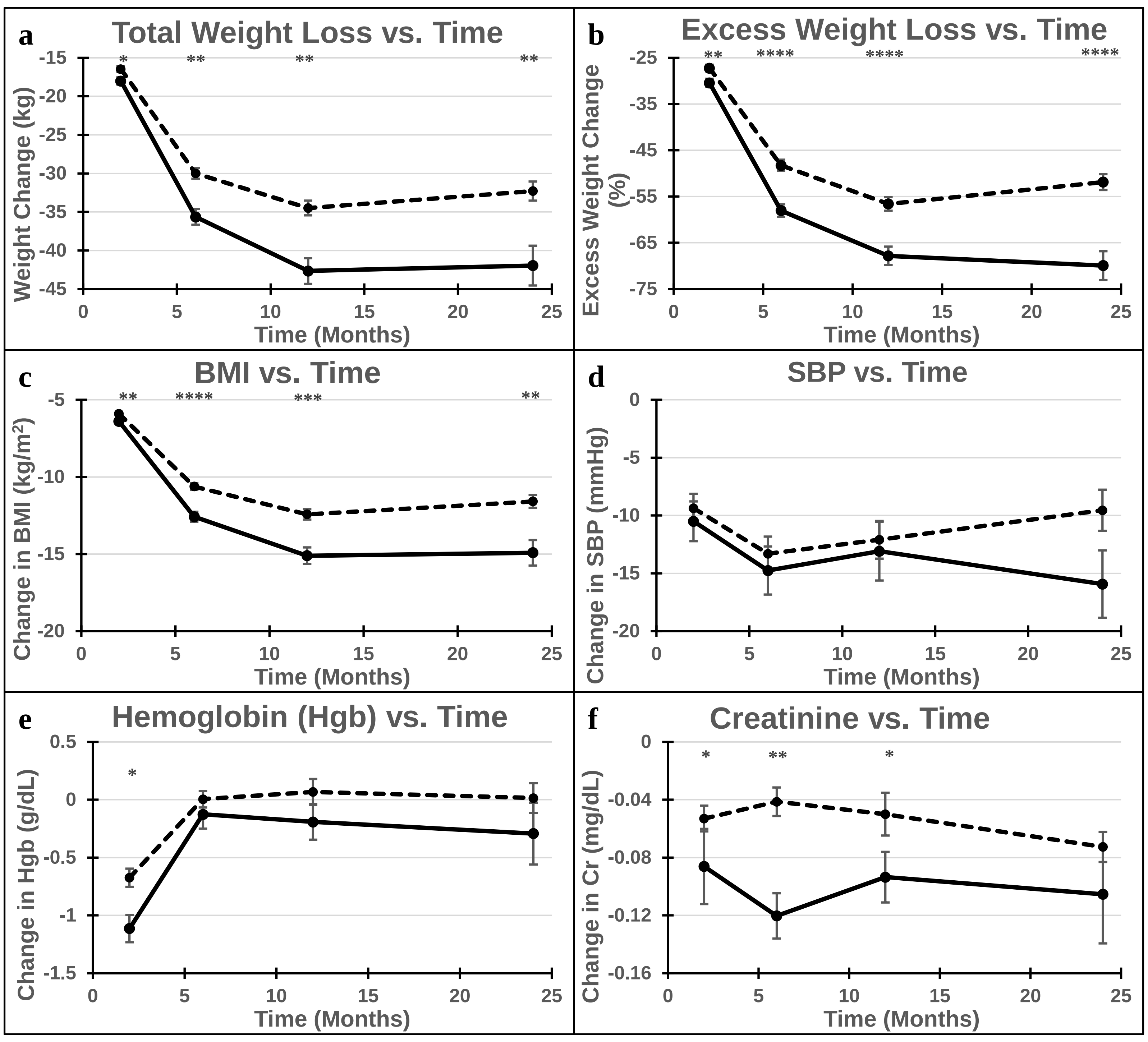

Supplementary Figure 2. Comparison of metabolic outcomes of sleeve gastrectomy (SG, dotted line) vs. Roux-en-Y gastric bypass (GB, solid line) performed after December 2010 at which point a more similar number of each procedure was being performed and baseline weights are not significantly different.. All graphs show the change in the specified variable over time. Error bars represent standard error of the mean. BMI, body mass index; SBP, systolic blood pressure; Hgb, hemoglobin; Cr, creatinine. For comparison between procedures: *, P < 0.05; **, P < 0.01; ***, P < 0.001; ****, P < 0.0001.

	GB Mean (SD)	GB n	SG Mean (SD)	SG n	p-value
Female (%)	77.8	153	79.6	274	0.19
Age (years)	43.6 (611.9)	153	45.0 (10.8)	274	0.22
Weight (kg)	134.9 (27.5)	153	136.8 (27.3)	274	0.49
BMI (kg/m^2)	47.6 (8.0)	153	48.6 (8.3)	274	0.20
Systolic BP (mmHg)	136.5 (18.4)	153	136.4 (18.1)	257	0.95
Hemoglobin A1C (%)	6.7 (1.4)	66	6.7 (1.3)	99	0.87
Vitamin D (ng/mL)	30.5 (16.1)	121	29.9 (14.1)	238	0.73
Vitamin B12 (pg/mL)	514.0 (218.7)	48	615.5 (302.9)	76	0.03
Hemoglobin (g/dL)	13.3 (1.3)	153	13.3 (1.2)	270	0.99
Creatinine (mg/dL)	0.84 (0.19)	151	0.82 (0.19)	270	0.23
Total Cholesterol (mg/dL)	174.1 (34.0)	16	176.03 (35.5)	37	0.85

Supplementary Table 1. Baseline characteristics of patients undergoing Roux-en-Y gastric bypass (GB) and sleeve gastrectomy (SG) after December 2010 at which point a more similar number of each procedure was being performed and baseline weights were not significantly different.
